# Pseudomonas aeruginosa keratitis in a tertiary referral center: a decade of clinical insights and therapeutic challenges

**DOI:** 10.1186/s12348-025-00538-x

**Published:** 2025-10-14

**Authors:** Mohammad Soleimani, Alireza Razavi, Sadra Jalali Najafabadi, Fatemeh Mahmoudi, Alireza Hayati, Melika Arab Bafrani, Hassan Asadigandomani

**Affiliations:** 1 Department of Ophthalmology, The University of North Carolina at Chapel Hill, Chapel Hill, NC 27599 USA; 2 Ocular Trauma and Emergency Department, Farabi Eye Hospital, Tehran University of Medical Sciences, Tehran, Iran; 3 Research Center for Clinical Virology, Tehran University of Medical Sciences, Tehran, Iran; 4 Department of Ophthalmology, Isfahan Eye Research Center, Isfahan University of Medical Sciences, Isfahan, Iran; 5 Students’ Research Committee (SRC), Qazvin University of Medical Sciences, Qazvin, Iran

**Keywords:** Pseudomonas aeruginosa, Bacterial keratitis, Antibiotic resistance, Endophthalmitis, Therapeutic keratoplasty

## Abstract

**Purpose:**

To evaluate the clinical characteristics, risk factors, antimicrobial resistance patterns, and outcomes of *Pseudomonas aeruginosa* keratitis in patients referred to a tertiary eye care center over a ten-year period.

**Methods:**

In this retrospective study, medical records of patients with culture-confirmed *Pseudomonas aeruginosa* keratitis between 2012 and 2022 at Farabi Eye Hospital, Tehran, were reviewed. Data collected included demographic and clinical features, antimicrobial susceptibility results, treatment modalities, and outcomes up to six months of follow-up.

**Results:**

A total of 172 patients were included. The mean age was 52.33 ± 22.01 years, with a nearly balanced gender distribution (49.42% male, 50.58% female). Trauma was the most common predisposing factor (30.23%). Corneal perforation occurred in 23.84% and endophthalmitis in 13.95% of patients. Surgical intervention was required in 68.6% of patients, with penetrating keratoplasty (PKP) performed in 35.5%. Advanced age, worse baseline best corrected visual acuity (BCVA), diabetes mellites, and corneal perforation were significantly associated with poorer visual outcomes. Antimicrobial susceptibility testing revealed preserved sensitivity to amikacin (95.93%), ciprofloxacin (95.93%), ceftazidime (95.35%), and gentamicin (92.44%), while high resistance rates were noted for chloramphenicol (48.26%) and cefazolin (59.30%).

**Conclusion:**

*Pseudomonas aeruginosa* keratitis remains a severe, sight-threatening condition associated with significant surgical burden and suboptimal visual outcomes. Despite retained sensitivity to key antibiotics, high rates of corneal perforation and endophthalmitis emphasize the need for timely diagnosis and aggressive management. Future prospective, multicenter studies with longer follow-up are warranted to better define long-term outcomes and guide treatment strategies.

## Background

Corneal blindness remains a significant public health concern worldwide, ranked among the leading causes of irreversible vision loss. According to the World Health Organization (WHO) reports, corneal opacity is the fifth leading cause of blindness globally (accounting for 3.2% of cases) [1]. Infectious etiologies are a major contributor to this burden. Trachoma-induced scarring alone blinds approximately 2 million people, and microbial keratitis is estimated to cause an additional 1.5–2.0 million new cases of monocular blindness each year [2]. Indeed, infectious keratitis is now recognized as a leading cause of corneal blindness worldwide. Its incidence varies widely by region (from a few cases per 100,000 population annually in some developed countries to several hundred per 100,000 in parts of South Asia), with a markedly higher disease burden in low- and middle-income communities [3]. The resulting socioeconomic and quality-of-life implications underscore the global importance of microbial keratitis as a cause of preventable blindness [1].

*Pseudomonas aeruginosa* merits special attention due to its prevalence and virulence among the diverse pathogens in microbial keratitis. *Pseudomonas aeruginosa* is frequently reported as one of the most common causative organisms in bacterial keratitis across many regions [4]. For example, epidemiological studies indicate that *Pseudomonas* accounts for roughly 6–39% of bacterial keratitis cases in the United States and about 8–21% in South Asia [5]. A key risk factor driving *Pseudomonas aeruginosa* keratitis is contact lens wear, which in developed countries has been associated with a 10- to 80-fold increased risk of corneal infection [3]. With an estimated 140 million contact lens users worldwide, contact lens–related *Pseudomonas* keratitis represents a substantial and growing clinical problem [3]. By contrast, in many low-income settings where lens use is less prevalent, corneal trauma with environmental contamination is a more common precipitant of bacterial keratitis [6]. *Pseudomonas aeruginosa* thrives as a ubiquitous organism in soil and water, and it has been identified as a leading pathogen following agricultural injuries in certain regions [7]. Thus, whether introduced via a contaminated contact lens or a corneal abrasion in rural areas, *Pseudomonas* is a formidable cause of keratitis in urban and rural populations.

Clinically, *Pseudomonas aeruginosa* keratitis is notorious for its aggressive course and therapeutic challenges. Infections caused by *Pseudomonas aeruginosa* often progress rapidly, with the bacterium’s proteases and toxins causing corneal liquefaction and melting; if untreated, complete corneal destruction can occur within 24–48 h of onset [8]. Patients typically present with severe suppurative keratitis (dense stromal infiltrates, hypopyon, and risk of perforation), and initial Management requires intensive topical antibiotic therapy (often fortified dual agents) administered at frequent intervals [9]. Despite modern antibiotics, outcomes can be guarded; even when the infection is eradicated, residual corneal scarring often impairs vision, and emergent surgical intervention is sometimes required [10, 11]. In tertiary referral settings, up to 20–50% of severe bacterial keratitis cases have been reported to necessitate at least one surgical procedure, such as therapeutic keratoplasty, to restore ocular integrity [12, 13]. Furthermore, the emergence of drug-resistant *Pseudomonas aeruginosa* strains has begun to complicate the therapeutic landscape, with increasing reports of multi-drug-resistant keratitis that prove difficult and costly to treat [14, 15]. Such factors highlight the substantial clinical impact of *Pseudomonas aeruginosa* keratitis and the imperative to optimize its Management.

In recent years, advances in molecular diagnostic technologies have expanded the tools available for detecting ocular pathogens beyond conventional culture. Shotgun sequencing has emerged as a hypothesis-free approach that can identify complete taxonomic and functional profiles of pathogens from very small DNA samples, offering higher sensitivity compared to standard PCR or histology [16]. Similarly, metagenomic next-generation sequencing (mNGS) has been applied in culture-negative microbial keratitis, enabling identification of previously undetected bacterial and viral agents and providing clinically meaningful guidance in challenging cases [17]. Moreover, mNGS-based 16 S rRNA sequencing has been utilized to characterize the ocular surface microbiome, revealing compositional differences between keratoconus patients and healthy controls, and underscoring the potential role of microbiota alterations in corneal disease [18]. Together, these evolving technologies highlight the growing importance of genomic and metagenomic approaches in the diagnosis, pathogen profiling, and pathophysiologic understanding of ocular infections, complementing traditional microbiological methods.

Considering the global burden and the challenges posed by *Pseudomonas aeruginosa* keratitis, the present study was undertaken to deepen understanding of this condition in a high-volume referral setting. We report a retrospective analysis spanning ten years of *Pseudomonas aeruginosa* keratitis patients at Farabi Eye Hospital in Tehran, Iran, one of the country’s largest tertiary ophthalmology centers. This decade-long review examines the clinical characteristics, risk factors, management strategies, and outcomes in a large cohort of patients with culture-proven *Pseudomonas aeruginosa* keratitis. By leveraging a decade of clinical insights from a tertiary referral center, our study aims to delineate patterns and outcomes of *Pseudomonas aeruginosa* corneal ulcers to inform future care protocols and improve management strategies for this sight-threatening infection.

## Methods

### Study design and setting

This retrospective study was performed at Farabi Eye Hospital, Tehran, Iran. Medical records of patients diagnosed with culture-confirmed *Pseudomonas aeruginosa* keratitis between 2012 and 2022 were reviewed. Collected data included demographic characteristics (age, gender), clinical features (affected eye, history of keratoplasty, comorbidities such as diabetes mellitus, hypertension (HTN), coronary artery disease (CAD), smoking, addiction, and contact lens use), clinical presentation details, visual acuity measurements, microbiological findings, therapeutic interventions, and final clinical outcomes. The study’s inclusion criteria were a confirmed diagnosis of *Pseudomonas aeruginosa* keratitis through culture and a minimum of six months of clinical follow-up. Patients who did not provide informed consent or had incomplete medical records were excluded.

Corneal samples were obtained using a sterile scraping technique under local anesthesia with 0.5% proparacaine. Corneal samples were cultured on chocolate blood agar and incubated at 35 °C. Gram staining and standard biochemical tests confirmed the identification of *Pseudomonas* species. Corneal samples were cultured on at least two occasions to avoid contamination and ensure accurate identification of pathogens. Consistent isolation of *Pseudomonas aeruginosa* from multiple cultures confirmed its role as the causative pathogen. An in vitro antibiotic susceptibility test was conducted using the disk diffusion method in accordance with Clinical Laboratory Standards Institute (CLSI) guidelines [19].

Indications for patient admission included central corneal involvement, infiltration size greater than 2 mm, evidence of corneal thinning or perforation, or the patient’s inability to attend regular daily outpatient follow-up visits. For the first 48 h, the initial antibiotic regimen consisted of hourly administration of fortified antibiotic eye drops, which was subsequently modified according to clinical response and antibiotic susceptibility results. Patients were monitored daily by experienced cornea subspecialists, each with at least five years of clinical and surgical expertise. Surgical interventions such as cyanoacrylate glue application, penetrating keratoplasty (PKP), tarsorrhaphy, amniotic membrane transplantation (AMT), or evisceration were performed as necessary in cases unresponsive to medical treatment.

Visual acuity was recorded using Snellen charts and converted to LogMAR for analysis. For severe visual impairment where visual acuity was less than 1/10, alternative scales such as counting fingers (CF), hand motion (HM), light perception (LP), and no light perception (NLP) were employed, and corresponding LogMAR values of 2.1, 2.4, 2.7, and 3.0 were assigned respectively [20].

### Ethical consideration

The Ethics Committee in Biomedical Research of Tehran University of Medical Sciences approved this study (IR.TUMS.FARABIH.REC.1400.074). All patients provided written informed consent permitting the use of their clinical data for research purposes. Patient-identifying details were anonymized through coding to maintain confidentiality and privacy. Access to patient data was limited exclusively to the primary researcher and authorized data collectors.

### Statistical analysis

Continuous variables were assessed for normality using Kolmogorov-Smirnov and Shapiro-Wilk tests. Variables with normal distribution were expressed as mean ± standard deviation (SD), whereas non-normally distributed data were expressed as median ± interquartile range (IQR). Qualitative variables were presented as frequency and percentage. Non-parametric tests, including the Mann-Whitney test, were employed to compare groups for continuous data. Fisher’s exact and Chi-square tests were used to compare categorical variables. Multinomial logistic regression was utilized to predict outcomes involving nominal variables with more than two unordered categories. Data analysis was conducted using SPSS software (version 26.0, SPSS Inc., Chicago, IL, USA), with statistical significance defined at a P-value less than 0.05.

## Results

Finally, one hundred seventy-two patients diagnosed with *Pseudomonas aeruginosa* keratitis were included. The average age was 52.33 ± 22.01 years (ranging from 20 to 92 years). The gender distribution among the patients was nearly balanced, with the right eye being the most frequently affected, occurring in 56.4% of patients. Comorbidities included HTN in 23.26% of patients and diabetes mellitus in 14.53%. Hypopyon was identified in 62% of cases, while 13.95% were diagnosed with endophthalmitis. The duration of hospitalizations varied; however, most patients (68.02%) required only a single hospital stay. The patients’ demographic and clinical details are outlined in Table 1.

**Table 1:** The demographics and clinical characteristics of participants at baseline

Variables	Sub-groups	Number (percentage%); Mean ± SD
Gender	Male	85 (49.42%)
	Female	87 (50.58%)
Age (year)	< 50	85 (49.42%)
	≥ 50	87 (50.58%)
Involved Eye	Right eye	97 (56.40%)
	Left eye	72 (41.86%)
	Both eye	3 (1.74%)
Comorbidities	Hypertension	40 (23.26%)
	Diabetes mellitus	25 (14.53%)
	Coronary artery diseases	23 (13.37%)
	Addiction	21 (12.21%)
	Smoking	21 (12.21%)
Hypopyon	No	66 (38.37%)
	Yes	106 (62%)
Endophthalmitis	No	148 (86.05%)
	Yes	24 (13.95%)
Corneal perforation	No	131 (76.16%)
	Yes	41 (23.84%)
Risk factors	History of trauma	52 (30.23%)
	History of contact lens wear	23 (13.37%)
	History of keratoplasty	13 (7.56)
Number of hospitalizations	1	117 (68.02%)
	2	23 (13.37%)
	3	13 (7.56%)
	4	6 (3.49%)
	5	3 (1.74%)
	6	3 (1.74%)
	7	3 (1.74%)
	8	1 (0.58%)
	11	1 (0.58%)
	17	1 (0.58%)
	25	1 (0.58%)
BCVA (LogMAR)	Pre	2.01 ± 0.96
	Post 6 months	1.92 ± 1.02

Regarding initial medications, the most frequently utilized option was the combination of fortified Amikacin + Cefazolin (87.21%). The remaining primary (empiric) and secondary medications used during treatment are summarized in Table 2.

**Table 2:** Primary (empiric) and secondary medications

Primary (empiric) medication	Number (percentage%)
Amikacin + Cefazolin	150 (87.21%)
Ciprofloxacin	17 (9.88%)
Levofloxacin	5 (2.91%)
Amphotericin B	1 (0.58%)
Itraconazole	1 (0.58%)
Voriconazole	1 (0.58%)
Tab Doxycycline	87 (50.58%)
Tab Acyclovir	2 (1.16%)
Tab Ciprofloxacin	1 (0.58%)
Secondary medication	
Amikacin + Ceftazidime	64 (37.21%)
Vancomycin + Ceftazidime	45 (26.16%)
Ciprofloxacin	17 (9.88%)
Levofloxacin	14 (8.14%)
Vancomycin	11 (6.39%)
Chloramphenicol	5 (2.91%)
Ofloxacin	5 (2.91%)
Amikacin + Ceftazidime	3 (1.74%)
Betamethasone	3 (1.74%)
Amikacin + Cefazolin	2 (1.16%)
Natamycin + Voriconazole	1 (0.58%)
Tab Doxycycline	19 (11.05%)
Tab Acyclovir	3 (1.74%)
Cap Cephalexin	1 (0.58%)

Table 3 provides an overview of the surgical procedures performed on patients with *Pseudomonas aeruginosa* keratitis. A significant majority of patients (68.6%) needed surgical interventions (Table 3).

**Table 3:** Surgical procedures used for *Pseudomonas aeruginosa* keratitis patients

Procedure	Repetition of procedure	Number (per-percentage%)
Tarsorrhaphy	14	1 (0.58%)
	7	1 (0.58%)
	4	3 (1.74%)
	3	3 (1.74%)
	2	4 (2.33%)
	1	27 (15.7%)
Cyanoacrylate glue + therapeutic contact lens	4	1 (0.58%)
	2	11 (6.4%)
	1	40 (23.26%)
Amniotic membrane transplantation	4	3 (1.74%)
	2	6 (3.49%)
	1	11 (6.40%)
*Penetrating keratoplasty*	5	1 (0.58%)
	4	1 (0.58%)
	3	2 (1.16%)
	2	4 (2.33%)
	1	53 (30.81%)
*Eye evisceration*	1	9 (5.23%)

Table 4 presents the results of in vitro antimicrobial sensitivity testing for various antibiotics. Amikacin and Ciprofloxacin demonstrate the highest sensitivity rates, recorded at 95.93%, indicating their strong effectiveness. Conversely, antibiotics such as Cefazolin (59.30%), Chloramphenicol (48.26%), and Trimethoprim/Sulfamethoxazole (20.93%) reveal significant resistance rates (Table 4).

**Table 4:** In vitro antimicrobial sensitivity results

Antibiotic	Sensitive number (per-percentage%)	Resistant number (per-percentage%)	Intermediate number (per-percentage%)
Amikacin	165 (95.93%)	3 (1.74%)	0 (0%)
Ciprofloxacin	165 (95.93%)	5 (2.91%)	1 (0.58%)
Ceftazidime	164 (95.35%)	3 (1.74%)	1 (0.58%)
Gentamicin	159 (92.44%)	4 (2.33%)	0 (0%)
Levofloxacin	142 (82.55%)	7 (4.07%)	4 (2.33%)
Imipenem	137 (79.65%)	4 (2.33%)	0 (0%)
Piperacillin/tazobactam	34 (19.77%)	0 (0%)	0 (0%)
Ofloxacin	18 (10.47%)	1 (0.58%)	0 (0%)
Tobramycin	7 (4.07%)	0 (0%)	0 (0%)
Moxifloxacin	4 (2.33%)	1 (0.58%)	0 (0%)
Cefazolin	2 (1.16%)	102 (59.30%)	0 (0%)
Norfloxacin	1 (0.58%)	0 (0%)	0 (0%)
Carbenicillin	1 (0.58%)	0 (0%)	0 (0%)
Ceftizoxime	1 (0.58%)	0 (0%)	0 (0%)
Chloramphenicol	1 (0.58%)	83 (48.26%)	0 (0%)
Trimethoprim/Sulfamethoxazole	1 (0.58%)	36 (20.93%)	0 (0%)
Vancomycin	0 (0%)	5 (2.91%)	0 (0%)
Cefixime	0 (0%)	2 (1.16%)	0 (0%)
Cefotaxime	0 (0%)	1 (0.58%)	1 (0.58%)
Cefalexin	0 (0%)	1 (0.58%)	0 (0%)
Erythromycin	0 (0%)	1 (0.58%)	0 (0%)
Tetracycline	0 (0%)	1 (0.58%)	0 (0%)
Ceftriaxone	0 (0%)	0 (0%)	1 (0.58%)

Although cefazolin was included in the most frequently prescribed empiric regimen (amikacin + cefazolin, 87.21% of cases), in vitro testing demonstrated a high resistance rate to cefazolin (59.3%). The secondary medication patterns (Table 2) suggest that many patients were transitioned to more effective antibiotics such as ceftazidime, ciprofloxacin, and levofloxacin once susceptibility results became available. However, the exact timing of these changes, could not be determined from our retrospective dataset.

Polymicrobial infection was observed in 8 out of 172 patients (4.65%). Among the co-infecting organisms, gram-positive cocci were the most common, identified in 5 cases (2.91%), followed by gram-positive bacilli in 3 cases (1.74%).

Among the 24 patients diagnosed with endophthalmitis, all smear results from both anterior chamber (AC) and vitreous samples confirmed the presence of *Pseudomonas aeruginosa*. Additionally, cultures from both AC and vitreous samples were positive in 5 patients, with *Pseudomonas aeruginosa* identified in all these cultures.

The mean baseline baseline best corrected visual acuity (BCVA) of the patients was 2.01 ± 0.96 LogMAR, which decreased to 1.92 ± 1.02 LogMAR at the six-month follow-up (*P* < 0.001). In multivariate linear regression with six-month BCVA as the dependent variable, the model explained 46.4% of variance (R² = 0.464, *p* < 0.001). Significant predictors of poorer vision included: Older age (β = 0.26, *p* = 0.001), worse baseline BCVA (β = 0.48, *p* < 0.001), diabetes mellitus (β = 0.14, *p* = 0.037), and corneal perforation (β = 0.14, *p* = 0.033). Other tested variables (gender, HTN, CAD, hypopyon, history of corneal transplantation, and contact lens wear) were not independently associated with outcome.

The need for surgical interventions was significantly associated with age (*r* = 0.309, *P* < 0.001), CAD (*r* = 0.192, *P* = 0.012), and HTN (*r* = 0.224, *P* = 0.003). Patients with HTN underwent surgical interventions at a significantly higher rate than those without HTN (87.5% vs. 62.9%, *P* = 0.003). Similarly, patients with CAD were more likely to require surgery than those without CAD (91.3% vs. 65.1%, *P* = 0.012). Multivariate logistic regression identified corneal perforation as the strongest predictor of surgical intervention (OR 30.4, 95% CI 3.55–260.8, *p* = 0.002). Other factors, including age, gender, diabetes mellitus, HTN, CAD, hypopyon, corneal transplantation, and baseline BCVA, were not statistically significant in the adjusted model.

The overall incidence of endophthalmitis in our cohort was 13.9% (24/172 eyes). On univariate analysis, most demographic and clinical factors (gender, diabetes mellitus, HTN, CAD, history of contact lens wear, corneal perforation, and history of corneal transplantation) were not significantly associated with endophthalmitis (all *p* > 0.05). However, Age ≥ 50 years was strongly associated with higher risk of endophthalmitis (*p* = 0.001, OR 6.05, 95% CI 1.97–18.55). In the multivariate logistic regression model including age, gender, systemic comorbidities, and baseline BCVA, none of the variables remained statistically significant predictors of the outcome. A significant correlation was also observed between corneal perforation and diabetes mellitus (*r* = −0.153, *P* = 0.045), with perforation occurring more frequently in diabetic patients than non-diabetic patients (26.5% vs. 8.0%, *P* = 0.044).

## Discussion

This ten-year retrospective study offers a comprehensive evaluation of 172 patients of culture-proven *Pseudomonas aeruginosa* keratitis managed at a tertiary referral center. The majority of patients required hospitalization and intensive treatment, with surgical intervention necessary in over two-thirds of patients. Notably, corneal perforation occurred in nearly one-quarter of patients, and endophthalmitis developed in 13.9%, underscoring the potentially sight-threatening nature of this infection. Final visual outcomes remained poor in most cases, with a mean BCVA of 1.92 LogMAR at six months. Age, male gender, and comorbid conditions such as HTN and CAD were significantly associated with a higher likelihood of requiring surgery. While disease severity and outcomes varied, antimicrobial susceptibility testing revealed preserved sensitivity to key antibiotics—especially Amikacin, Ciprofloxacin, Ceftazidime, and Levofloxacin—supporting their continued use in empiric therapy.

One of the major strengths of this study is its relatively large sample size compared to similar reports from various countries. Our study represents one of the most extensive single-center investigations of *Pseudomonas aeruginosa* keratitis to date, surpassing the sample sizes reported in many prior studies across diverse regions including Iran [12], the United States [21], Europe [22], South America [23], India [24], and East Asia [25]. This robust sample size strengthens the reliability of our findings and supports broader generalizability of clinical patterns, risk factors, and treatment outcomes in *Pseudomonas aeruginosa* keratitis.

Age and gender distribution in our cohort appear to reflect the dominant predisposing factors for *Pseudomonas aeruginosa* keratitis in our region. In our study, trauma was the most common identified risk factor (30.2%), while contact lens use accounted for only 13.4% of cases. This contrasts with patterns in high-income countries, where contact lens wear is often the primary risk factor, frequently associated with younger, predominantly female patients [26, 27]. For example, Enzor et al. reported that patients with *Pseudomonas aeruginosa* keratitis who wore contact lenses had a mean age of 39.2 years, compared to 71.9 years among non–contact lens wearers [26]. In the same study, females predominated among contact lens users (61.3%), whereas the gender distribution was more balanced among non–lens users (52.9% female vs. 47.1% male); in our study, the near-equal gender distribution likely reflects the lower prevalence of contact lens use and the predominance of trauma as the main risk factor [26]. A similar pattern of male predominance and trauma as the leading risk factor was observed in the study from Romania [22]. These findings suggest that regional differences in socioeconomic status, lifestyle, and occupational exposure not only influence risk factors but also shape the demographic profile of *Pseudomonas aeruginosa* keratitis.

The incidence of corneal perforation in our cohort was notably high, occurring in 23.84% of cases. This figure is considerably elevated compared to similar studies. For instance, Enzor et al. reported a perforation rate of 9.8% in a cohort exclusively composed of patients with *Pseudomonas aeruginosa* keratitis, making their population directly comparable to ours [26]. In broader studies that analyzed bacterial keratitis regardless of the causative organism, the reported rates of perforation were generally lower: 19% in the study by Kusumesh et al. [28], 11.5% in the report by Jin et al. [29], and 10.8% in the series by Almulhim et al. [30]. These comparisons underscore the particularly severe disease burden observed in our population and may reflect later presentation or limited access to early ophthalmic care.

This severity is further highlighted by the exceptionally high rate of therapeutic PKP in our study. A total of 61 patients (35.5%) required one or more graft procedures, with 30.8% undergoing at least one keratoplasty and several needing repeated surgeries. This is substantially higher than the 6.5% rate of tectonic grafting reported by Enzor et al. in a similarly defined population of *Pseudomonas aeruginosa* keratitis [26]. Even in broader studies that included various bacterial pathogens, the proportion of patients requiring keratoplasty was typically lower—for instance, Das et al. reported a rate of 23.1% among 13,625 cases of non-viral microbial keratitis [31]. Taken together, these findings reinforce the aggressive clinical course and high surgical burden observed in our cohort.

In our cohort, the incidence of endophthalmitis was markedly high at 13.95%, which stands in stark contrast to previous reports. The reported incidence of endophthalmitis following microbial keratitis in the literature ranges from 0.29% to 6.1% [32–34]. Notably, these studies examined bacterial keratitis in general and did not distinguish by causative organism [32–34]. The significantly higher rate observed in our study may reflect the inherently aggressive nature of *Pseudomonas aeruginosa* infections [25], and the overall poorer outcomes in our patient population. On univariate analysis, age ≥ 50 years was associated with significantly higher odds of endophthalmitis; however, after multivariate adjustment for age, gender, systemic comorbidities, and baseline vision, no independent predictors remained significant. Compared with the series by Christopher et al. [35], which identified presenting vision of LP/NLP, history of cataract surgery, and full-thickness ulcer/perforation as important risk factors, our cohort did not show similar associations. These differences may be explained by pathogen-specific effects, since our study exclusively evaluated *Pseudomonas aeruginosa* keratitis, as well as referral bias in a tertiary care setting. Future research should specifically investigate endophthalmitis as a distinct outcome in *Pseudomonas aeruginosa* keratitis, given its alarming frequency and potential for severe visual morbidity.

Antibiotic susceptibility patterns of *Pseudomonas aeruginosa* vary significantly across regions, influenced by local prescribing practices, antibiotic availability, and infection control measures [25, 36, 37]. In our study, resistance to commonly used antibiotics remained relatively low: ciprofloxacin and gentamicin each showed resistance rates below 3%, and ceftazidime and amikacin resistance were similarly low at 1.74%. These findings are consistent with global reports from earlier decades, where ciprofloxacin and gentamicin resistance remained under 10% in most countries during the 1990–2003 period [25]. However, significant geographic variability has been documented. For instance, in a study from India covering the 1992–2003 period, resistance to ciprofloxacin reached 30%, and gentamicin resistance climbed to 46%, underscoring the importance of continuous regional surveillance [25, 36].

Our findings highlight a discrepancy between empiric treatment regimens and actual in vitro susceptibility patterns, particularly regarding cefazolin. While empiric use of cefazolin remains common in our setting, culture data indicate limited efficacy, underscoring the need to refine empiric protocols. Although secondary treatment data suggest that clinicians frequently adjusted therapy once susceptibility results became available, we were unable to quantify the exact timeliness of these adjustments due to the retrospective nature of our study. This represents an important limitation and reinforces the need for future prospective studies that track real-time decision-making in relation to culture results.

In contrast, beta-lactam antibiotics—particularly older-generation cephalosporins—demonstrated markedly reduced efficacy in our cohort. Cefazolin, for example, showed only 1.16% sensitivity, with over 59% of isolates being resistant. This finding aligns with literature describing beta-lactam resistance in nosocomial *Pseudomonas* infections, often mediated by mechanisms such as metallo-beta-lactamase production [38, 39]. Similar to our findings, the study by Nagaraju Konda et al. also reported high resistance rates to chloramphenicol among *Pseudomonas aeruginosa* isolates, underscoring its limited utility in the treatment of bacterial keratitis caused by this pathogen [24]. These results support the continued use of ciprofloxacin, ceftazidime, and amikacin as empiric therapy for *Pseudomonas aeruginosa* keratitis in our setting, while also highlighting the limited utility of first-generation cephalosporins and the need for stewardship policies tailored to local resistance profiles.

Compared with the long-term trend data reported in the study by Joseph et al., which documented a steady rise in antimicrobial resistance over three decades, our results indicate relatively preserved susceptibility [40]. In particular, ciprofloxacin resistance in our cohort remained below 3%, contrasting with the rising resistance rates reported in India, where ciprofloxacin resistance in gram-negative isolates increased from 11.5% to 18.7%. Similarly, while Joseph et al. reported a significant decline in susceptibility of gram-positive isolates to cefazolin (95.5% to 66%) and amikacin (88% to 55%), our *Pseudomonas* isolates retained high susceptibility to amikacin, with resistance rates below 2%. However, our study also demonstrated markedly reduced efficacy of older-generation beta-lactams such as cefazolin, underscoring the need for continued surveillance to monitor emerging resistance trends in ocular pathogens [40].

In our study, diabetes mellitus was significantly associated with a higher incidence of corneal perforation, suggesting that systemic metabolic status may influence keratitis severity. This observation is supported by findings from Almulhim et al., who similarly reported poorer clinical outcomes in diabetic patients with infectious keratitis [30]. Several studies have proposed that diabetes mellitus contributes to both structural and functional abnormalities of the cornea, impairing epithelial integrity and delaying wound healing [41, 42]. These changes increase the risk of secondary infection, prolong disease duration, and often reduce responsiveness to medical therapy. As a result, diabetic patients are more likely to require surgical intervention to control disease progression and preserve ocular integrity [42].

In our study, older age was significantly associated with worse visual outcomes at the 6-month follow-up, a higher likelihood of surgical intervention, and an increased incidence of endophthalmitis. These findings are consistent with previous research, including the study by Thamizhselvi et al., which reported poorer clinical outcomes in older patients with *Pseudomonas* keratitis [43]. Age-related physiological changes in the ocular surface—such as reduced corneal sensation and decreased subepithelial nerve density—may contribute to this vulnerability [44]. For example, Niederer et al. demonstrated a linear decrease in corneal nerve density of approximately 0.9% per year in healthy individuals [45]. This gradual decline in corneal innervation impairs epithelial maintenance, delays wound healing, and reduces the eye’s defense against microbial invasion [45].

Beyond host factors such as age, pathogen-related characteristics may also influence clinical outcomes. A study by Choy et al. highlighted the role of the type III secretion system (T3SS) genotype in determining prognosis [46]. In their cohort, invasive strains were more common in older males and patients with a history of ocular surgery or trauma, and were significantly associated with worse presenting and final visual acuity, larger and deeper infiltrates, and delayed epithelial healing. In contrast, cytotoxic strains were strongly linked to contact lens wear. Importantly, invasive strains were identified as independent poor prognostic factors for final vision and time to re-epithelialization, with a trend toward higher fluoroquinolone resistance. Taken together, these findings suggest that both host-related vulnerabilities (such as advanced age) and pathogen-specific virulence mechanisms (such as T3SS genotype) contribute to the unfavorable outcomes frequently observed in *Pseudomonas aeruginosa* keratitis [46].

This study has several limitations that should be acknowledged. First, its single-center design may limit the generalizability of the findings to broader populations with varying healthcare access and regional pathogen profiles. Second, the retrospective nature of data collection inherently introduces potential biases, including variability in documentation and loss of certain clinical details. Additionally, our center—being a national tertiary referral hospital located in the capital—serves patients from distant provinces. As a result, many patients returned to their hometowns for postoperative care, and consistent long-term follow-up beyond six months was not feasible for the majority of cases. Consequently, we were unable to evaluate late visual outcomes or long-term graft survival. These limitations highlight the need for future multicenter, prospective studies with standardized long-term follow-up protocols to better characterize the extended clinical course and therapeutic response of *Pseudomonas aeruginosa* keratitis across diverse populations.

## Conclusions

This ten-year retrospective analysis highlights the significant clinical burden of *Pseudomonas aeruginosa* keratitis in a tertiary referral setting, marked by high rates of corneal perforation, surgical intervention, and endophthalmitis. While antimicrobial susceptibility to key agents such as amikacin, ciprofloxacin, and ceftazidime remained favorable, the limited efficacy of chloramphenicol and older-generation cephalosporins was evident. Older age and systemic comorbidities, including HTN, diabetes mellitus, and CAD, were associated with worse clinical outcomes and increased surgical needs. Despite intensive treatment, final visual outcomes were suboptimal in many patients. Given the retrospective, single-center nature of this study and the absence of long-term follow-up—due to the referral-based structure and geographic dispersion of patients—our findings underscore the need for future prospective, multicenter studies with extended follow-up to better inform management strategies for this vision-threatening condition.

## Data Availability

The datasets used and/or analyzed during the current study are available from the corresponding author on reasonable request.

## Abbreviations

WHO: World health organization
mNGS: Metagenomic next-generation sequencing
HTN: Hypertension
CAD: Coronary artery disease
CLSI: Clinical laboratory standards institute
PKP: Penetrating keratoplasty
AMT: Amniotic membrane transplantation
CF: Counting fingers
HM: Hand motion
LP: Light perception
NLP: No light perception
SD: Standard deviation
IQR: Interquartile range
BCVA: Best-corrected visual acuity
AC: Anterior chamber
